# From descriptive to generative: foundation-model approaches for spatial transcriptomics

**DOI:** 10.1093/bib/bbag554

**Published:** 2026-10-08

**Authors:** Ting Fan, Yufei Leng, Jiatong Zhang, Zhichang Zhang

**Affiliations:** School of Intelligent Medicine, China Medical University, No. 77 Puhe Road, Shenbei New District, Shenyang, Liaoning Province, 110122, China; School of Stomatology, China Medical University, No. 77 Puhe Road, Shenbei New District, Shenyang, Liaoning Province, 110122, China; The First Clinical College, China Medical University, No. 77 Puhe Road, Shenbei New District, Shenyang, Liaoning Province, 110122, China; School of Intelligent Medicine, China Medical University, No. 77 Puhe Road, Shenbei New District, Shenyang, Liaoning Province, 110122, China

**Keywords:** spatial transcriptomics, multimodal integration, gene regulatory networks, single-cell interactions, histological images, large language models

## Abstract

Spatial transcriptomics has advanced the study of biological systems by facilitating the mapping of gene expression within native tissue environments. Nevertheless, the intrinsic high dimensionality, pronounced data sparsity, and intricate nonlinear relationships between tissue morphology and the transcriptome pose challenges for conventional analytical frameworks in capturing long-range biological dependencies. This manuscript introduces a unified conceptual framework, termed “cells as words, tissues as sentences,” for examining how the contextual learning and generative capabilities of large language models (LLMs) and other foundation-model approaches can be applied to characterize the spatial organization of tissues. We synthesize evidence from published studies showing that Transformer-based LLMs, Mamba/state-space models, and diffusion-based models have been applied to tasks including spatial domain identification, cell–cell interaction inference, and cross-modal alignment. This shift from descriptive analysis toward generative modeling supports reconstruction and prediction tasks, while highlighting the need for rigorous validation of perturbational and spatiotemporal inference. Ultimately, this framework provides a systematic perspective for integrating spatial information and evaluating the demonstrated capabilities and limitations of foundation models, while identifying opportunities for generating testable hypotheses about tissue organization and disease progression.

## Graphical Abstract

**Graphical Abstract:**
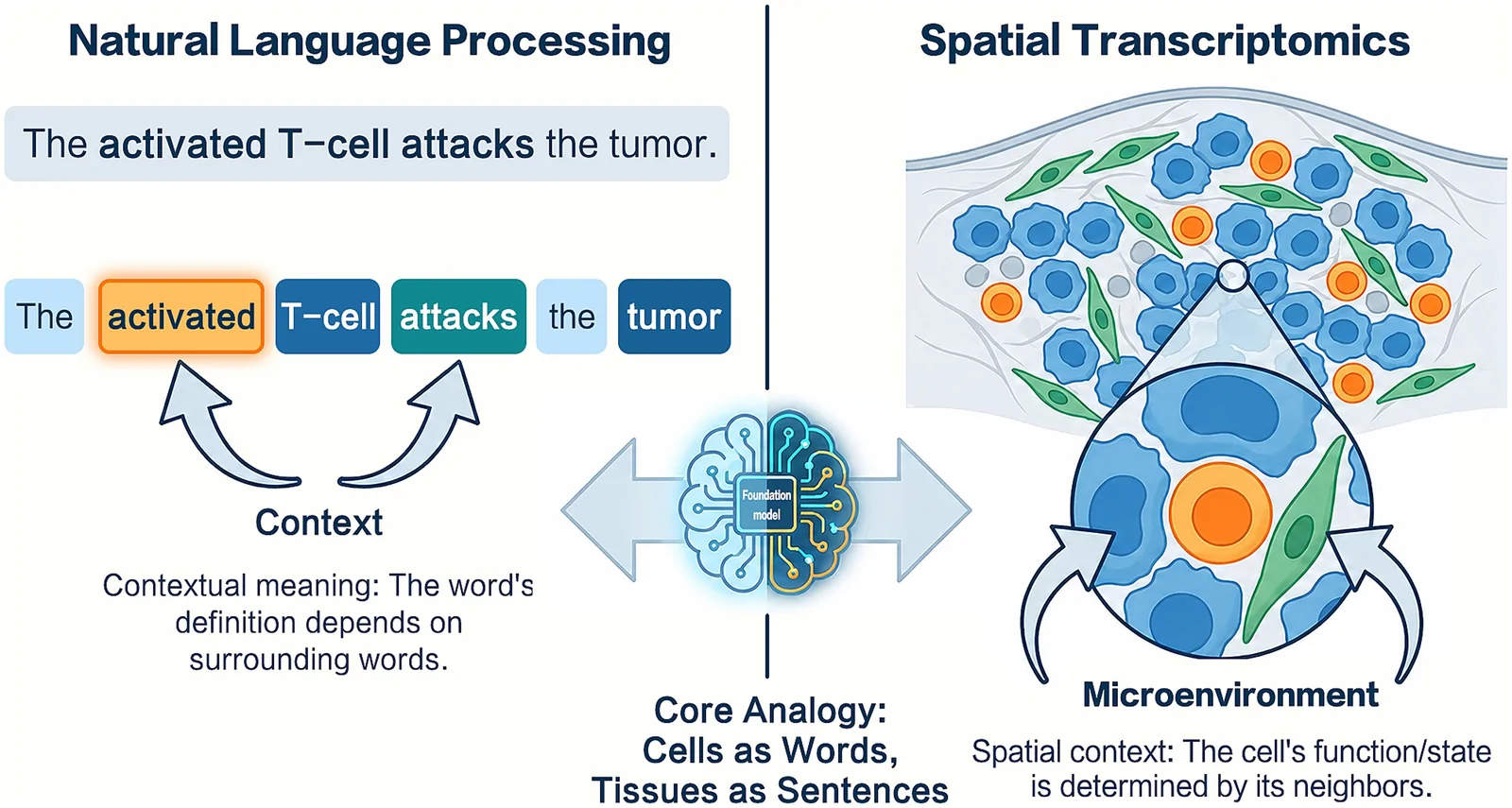

## Introduction

Spatial transcriptomics (ST) facilitates the high-throughput mapping of gene expression within native tissue environments, maintaining the spatial context that is typically lost in dissociated single-cell sequencing. As ST technologies progress toward higher resolution and multimodal profiling, the resultant datasets have become increasingly high-dimensional, sparse, and heterogeneous, presenting novel challenges for computational analysis. While graph-based and spatially regularized methods effectively capture local neighborhood relationships, emerging sequence-based and foundation-model approaches offer complementary strategies for learning broader contextual representations and supporting data reconstruction and generation.

A useful conceptual analogy is to view spatial units in ST as tokens and the surrounding tissue as their context, motivating the phrase “cells as words, tissues as sentences.” Related token-based representations have already been used in biological foundation models. For example, Geneformer represents genes as tokens and uses their ranked expression context to model cellular states [1]. This provides a methodological precedent for treating biological entities as tokens and modeling their contextual relationships. However, Geneformer operates on single-cell transcriptomic profiles and does not establish “cells as words, tissues as sentences” as a formal framework for ST. We therefore do not claim that the general ideas of biological tokenization or context modeling are new. Instead, as detailed below, this review applies the analogy explicitly to the organization and comparison of foundation-model approaches in ST. We emphasize that this is a conceptual analogy rather than a claim of direct biological or architectural equivalence. Unlike words in a sentence, spatial units do not possess a unique linear order, and their relationships are inherently two- or three-dimensional. Moreover, the relevant spatial unit depends on platform resolution: a token may represent a multicellular spot, an individual cell, or a subcellular measurement. Thus, “cell” in the analogy serves as an intuitive abstraction rather than a universal computational unit. We use this analogy as a conceptual organizing principle rather than a formally established ST framework: tokenization defines the “words”, while spatial serialization organizes spatially related tokens into sequence-like contexts. This sequence-based view is complementary to, rather than a replacement for, graph- or coordinate-based representations.

In this review, we make a critical distinction between large language models (LLMs) in the strict sense and the broader category of foundation-model approaches. LLMs are generally understood to be large-scale sequence models, primarily based on the Transformer architecture, and are trained using language-modeling objectives such as masked-token prediction or autoregressive prediction. In contrast, models such as Mamba and other state-space models (SSMs) employ alternative sequence-processing mechanisms, diffusion models are trained through iterative denoising processes, and vision-language or multimodal models integrate heterogeneous modalities. Given the differences in architecture and learning objectives among these approaches, we adopt the term “foundation-model approaches” to encompass this broader category, rather than classifying them all under the singular category of LLMs.

Within this broader context, we employ the metaphor “cells as words, tissues as sentences” as a conceptual framework to systematically organize three fundamental components: gene tokenization, spatial serialization, and self-supervised or generative learning objectives. We critically examine their implementation across various model architectures, including Transformer-based sequence models, Mamba/SSMs, diffusion-based generative models, and vision-language or multimodal foundation models. Rather than introducing a novel architecture or algorithm, the primary contribution of this review is to offer a cohesive perspective that relates spatial resolution, representation, context, and downstream tasks across diverse ST settings. We categorize representative studies according to major biological tasks, such as spatial domain identification, expression reconstruction, perturbation-response prediction, cell–cell interaction analysis, and cross-modal alignment. Consequently, this framework serves as a taxonomy and synthesis of evidence, rather than an experimentally validated unified model.

In contrast to existing reviews that predominantly address ST methods, multimodal integration, or foundational models as distinct subjects, this review offers a task-oriented framework that interlinks model representation and architecture with biological applications and validation. Specifically, we analyze the roles of tokenization, spatial context representation, and generative objectives in facilitating key ST tasks; compare the computational principles and applicability of various model families; and critically assess limitations such as hallucination, spatial data leakage, cross-domain generalization, scalability, and biological validation. Together, this perspective aims to clarify how emerging foundation-model approaches can support the transition from descriptive analysis toward context-aware and generative modeling in ST. The overall organization of this review—from gene tokenization and spatial serialization to model architectures, pretraining objectives, and downstream biological tasks—is illustrated in Fig. 1.

**Figure 1:**
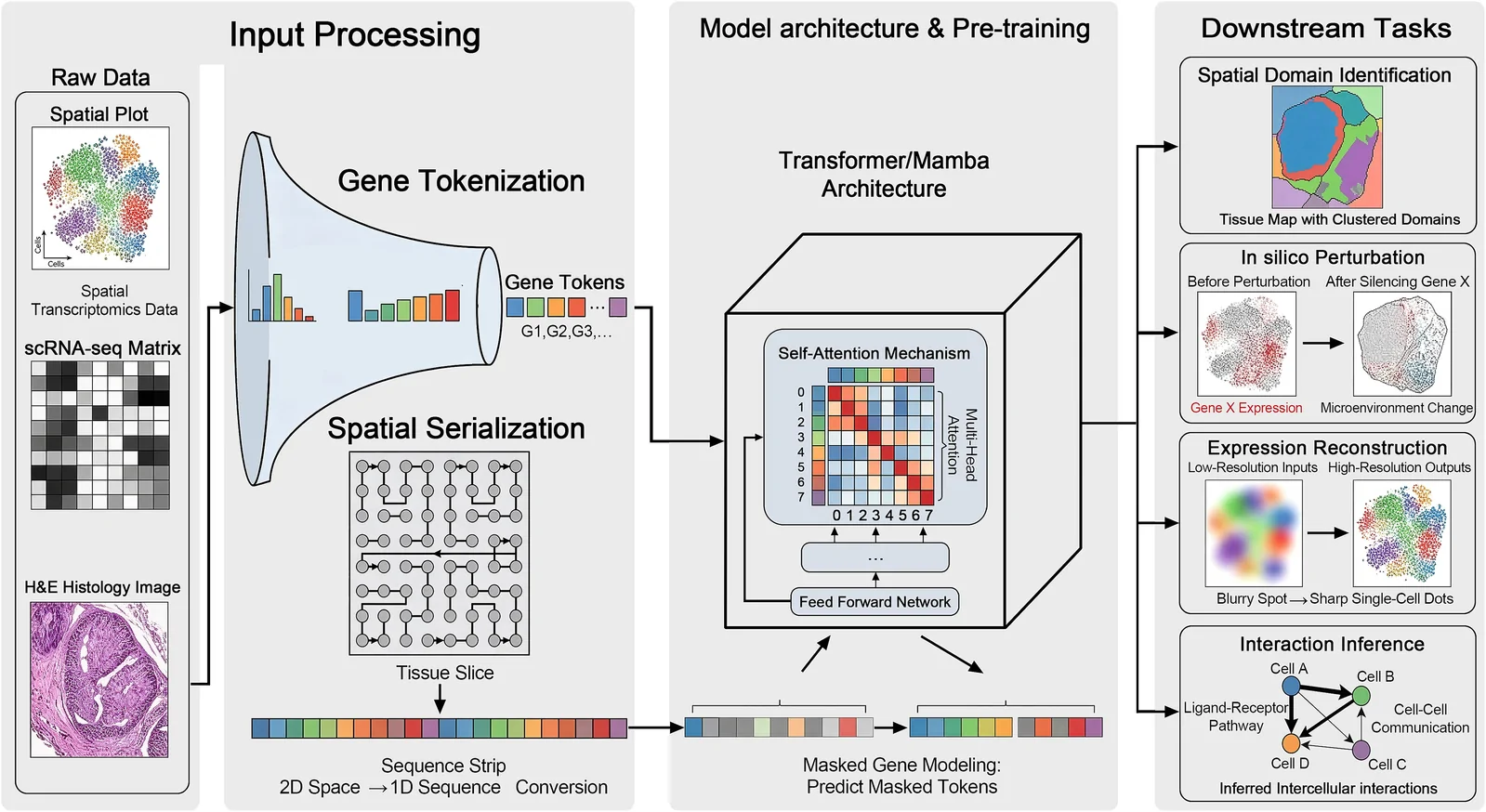
Conceptual pipeline of foundation-model approaches for ST under the “cells as words, tissues as sentences” framework. Left: input processing, in which gene expression values are converted into model-readable gene tokens (gene tokenization) and two-dimensional tissue topology is converted into one-dimensional sequences (spatial serialization). Middle: model architecture and pretraining, in which Transformer or state-space (Mamba) architectures with self-attention are trained with MGM. Right: representative downstream biological tasks, including spatial domain identification, virtual (*in silico*) perturbation, super-resolution, and cell–cell interaction inference.

## Computational principles in the biological context

### Context dependency

LLMs demonstrate the ability to use contextual information to resolve semantic ambiguity, a principle that parallels the interpretation of cellular states in ST. Cellular plasticity indicates that the phenotype and transcriptional state of a cell are influenced by factors such as the microenvironment, spatial location, and neighboring cells. Consequently, cell identity cannot always be inferred from intrinsic expression profiles alone and should be interpreted within its spatial context. We note that context dependence does not imply that cells fully revert to their original state once the surrounding context is removed; direct evidence for such reversibility remains limited, and we therefore interpret context-dependent states cautiously. This principle is particularly evident in spatially heterogeneous solid tumors. In colorectal and breast cancers, epithelial cells located in the tumor core and invasive margin display distinct gene expression programs [2], suggesting that cells of similar lineage may adopt different functional states depending on their local niche.

As a result, ST analysis is increasingly evolving from simple isolated transcriptional comparisons to a more comprehensive approach that includes the integrated modeling of cellular neighborhoods, spatial topology, and expression profiles for an in-depth characterization of cell states. The incorporation of spatial context enables the distinction of cell populations that, despite being phenotypically similar, display unique functional characteristics. In invasive ductal breast carcinoma, for instance, such strategies can identify malignant subpopulations enriched at the tumor margin and associated with increased metastatic potential [3]. Integrating local neighborhood structure with global spatial features further enables the dissection of state differences between similar cell types in tumor core and tumor edge regions, while revealing putative regulators associated with region-specific transitions [4]. These findings suggest that spatial context provides an important complement to intrinsic transcriptional information for characterizing cell states in complex tissues.

### Biological interpretation of attention mechanisms

Attention mechanisms (AMs) provide a way to selectively weight information from different features or spatial units during model inference. In ST, AMs can integrate spatial relationships with expression features and thereby contribute to representation learning. In contrast to conventional correlation-based methods, which generally assess global or pairwise associations, attention weights are dynamically derived from input representations and can be tailored to the spatial and molecular context relevant to each prediction task. By assigning varying degrees of importance to input relationships, AMs enable models to prioritize information relevant to a specific prediction task. Nonetheless, these weights should not be directly interpreted as biological interactions or regulatory relationships. Their biological meaning depends on the model architecture, input representation, and independent validation.

In the analysis of cell–cell signaling, AMs integrate spatial proximity with expression context, better reflecting the biological fact that cellular communication depends on the local tissue environment. Accordingly, models incorporating spatial adjacency and cell interaction information can further map ligand–receptor-mediated communication networks [5]. In gene co-expression analysis, AMs overcome the limitations of traditional global correlation methods, allowing local coordinated expression patterns specific to particular spatial regions or cell states to emerge. Relevant studies have shown that attention weights can highlight gene associations closely related to spatial reconstruction [6] and identify key gene interaction patterns linked to tumor invasiveness [7]. These results support the use of attention as a representation tool, while biological interpretation still requires independent evidence.

### From discriminative to generative models for imputation and gene regulatory network inference

A notable limitation of ST is the inherent sparsity present in expression matrices. This sparsity is characterized by a high proportion of zero or undetected gene expression measurements, which result from limited transcript capture, insufficient sequencing depth, and low transcript abundance. Although both discriminative and generative methods are capable of learning from observed signals, generative models possess the additional capability to reconstruct unobserved expression states using available contextual information. Achieving accurate reconstruction may necessitate the integration of gene co-expression patterns, spatial organization, and cell–cell interactions, as these factors offer complementary constraints on plausible expression states. Recent studies have shown that such models can preserve cancer-associated co-expression while supporting gene regulatory network inference [8], and can recover layer-specific cell–cell communication after integrating single-cell RNA sequencing (scRNA-seq) with ST data [9].

This link becomes stronger in multimodal settings. By combining histology, spatial coordinates, and scRNA-seq data, generative models can reconstruct missing expression within a richer biological context. Such reconstructions may better reflect tissue morphology and molecular structure, and reported studies have explored downstream effects of mutated genes [10] and the activity of core gene regulatory networks (GRNs) pathways [11]. Generative modeling in ST should therefore be viewed as extending imputation toward context-aware reconstruction, while stronger claims about regulatory mechanisms require independent biological validation.

## Building biological foundation models: from tokens to architectures

### Tokenization of input data

#### Discretizing high-dimensional continuous features: mapping gene expression profiles to tokens

Within the “cells as words” analogy, these tokenization strategies define how each “word”—the molecular identity of a spatial unit—is encoded, determining what biological information each token carries into the model. In foundational models for ST, tokenization converts the molecular profile of each spatial unit into a model-compatible representation. Depending on platform resolution, the spatial unit may correspond to a multicellular spot, an individual cell, or a subcellular measurement. The definitions of tokens, sequences, and spatial context at different platform resolutions are summarized in Table 3.

These resolution levels are not biologically interchangeable. Cell identity and cell–cell communication should not be inferred directly from spot-level measurements unless supported by deconvolution, cell-type mapping, or cellular-resolution validation. Common strategies for tokenization encompass continuous-value encoding, binning, and rank-based encoding. Continuous-value encoding involves the direct use of raw or normalized expression values, thereby maintaining detailed quantitative information. However, this approach is vulnerable to noise and batch effects. Binning involves mapping expression values into discrete intervals, thereby enhancing robustness while maintaining coarse expression gradients. Rank-based encoding represents genes by their relative expression order within each spot or cell, thereby mitigating the impact of absolute-scale variation.

These strategies preserve biological signals in different ways. Continuous-value encoding can capture subtle changes, but it is more easily affected by technical variation. For example, when principal component analysis is applied to continuous expression profiles from multi-platform human pancreatic datasets, samples often cluster by batch rather than by cell type [12]. Binning offers a more robust compromise. It preserves approximate expression hierarchies and reduces noise. More broadly, different tokenization strategies reflect different assumptions about which features of expression are most biologically informative. For example, CellMemory uses a scale-to-bin discretization scheme to reduce fluctuations in absolute expression caused by differences in sequencing depth. This shifts the focus from raw magnitude to relative expression tiers [13]. By contrast, the rank-based encoding used in Nicheformer is robust to batch effects and is also consistent with the biology of cell-state transitions. Cell-state changes are often driven not by isolated shifts in absolute transcript abundance, but by changes in the relative ordering of genes within regulatory cascades. By converting absolute expression into relative rank, this strategy helps preserve cellular identity even under large-scale perturbation [14]. In summary, encoding continuous values prioritizes quantitative accuracy, binning achieves a balance between structural expression and robustness, and rank-based encoding offers an improved equilibrium between cell type specificity and batch-to-batch consistency. These methodologies dictate the manner in which molecular information is encoded within individual tokens, while the interrelationships among tokens necessitate a distinct spatial-contextual representation.

#### Linearizing multidimensional topology: spatial serialization strategies for preserving microenvironmental context

Spatial serialization, the process of converting spatial measurements into an ordered sequence, is one strategy for adapting multidimensional ST measurements to one-dimensional sequence models [15]. In this process, spatial units in two- or three-dimensional tissue space are mapped into an ordered sequence. This conversion allows sequence-based models to process spatially structured ST data and model dependencies among spatially related units using sequence-processing mechanisms. However, tissues do not possess a unique biological ordering, and different traversal paths may produce different sequences. Serialization may therefore separate physically adjacent units or introduce artificial sequential neighbors. Its effectiveness depends on how well the chosen ordering preserves biologically meaningful spatial relationships [3, 16]. Current spatial serialization strategies include predefined paths, spatial ordinal serialization, and graph-based traversal. Predefined paths follow fixed rules, whereas spatial ordinal serialization orders cells according to their spatial organization while retaining some randomness, as in GeST [15]. Graph-based methods generate sequences by traversing spatially connected nodes.

The first type usually uses space-filling curves, such as Peano curves and Hilbert curves. These methods are easy to implement and follow a uniform rule. They can be useful for relatively regular spatial grids, such as those generated by 10× Visium, where the sampling geometry is more structured. However, in high-resolution data such as Slide-seqV2, fixed paths are more easily affected by irregular spatial distributions. This can weaken the preservation of local neighborhood relationships. These methods also have clear limits in heterogeneous tissue modeling, three-dimensional multi-section integration, and multimodal data integration [17]. By contrast, graph-based traversal methods usually build graphs with K-nearest neighbors or an r-radius strategy, while combining spatial proximity and expression similarity. Sequences are then generated by depth-first search or breadth-first search. These methods are better suited to irregular sampling, complex local relationships, and structural organization across sections or modalities. As a result, they can be useful for modeling heterogeneous ST data and capturing spatially dependent gene expression patterns [18]. As a concrete biological example, GeST was evaluated in an *in silico* ischemia perturbation experiment using a 10× Visium mouse-brain ischemia dataset comprising four ischemic and four sham-control sections (19 777 spots) together with the Mouse1 MERFISH dataset. After perturbing an infarct-core region with ischemia-associated differentially expressed genes (DEGs), GeST predicted the transcriptional response in the neighboring proximal peri-infarct area. It correctly classified 70.11% of 87 reference DEGs, compared with 44.8% for a naive baseline, and achieved a higher Pearson correlation with the reference expression profile [15]. In summary, spatial serialization extends beyond a mere technical transformation of spatial data into a one-dimensional format; it offers an organization akin to the “tissues as sentences” analogy. This approach embodies a modeling decision that balances sequence compatibility with the preservation of spatial topology. Alternatively, graph-based or coordinate-aware representations offer methods to maintain spatial relationships without enforcing a singular traversal path.

Serialization is notably characterized by its sensitivity to permutation, rotation, and traversal, whereby varying starting points, orientations, or traversal paths can yield disparate sequences from an identical tissue topology. This limitation is particularly pertinent to models that explicitly linearize spatial units via space-filling curves or graph traversal, as opposed to graph-native or coordinate-aware models which directly engage with spatial relationships. Equivariant representations offer an additional alternative by mitigating sensitivity to geometric transformations. For sequence-based models, robustness may be enhanced through the aggregation of multiple serialization paths or the integration of topology-preserving positional encodings, such as relative coordinates, pairwise distances, or neighborhood information. These strategies reduce the risk that biological conclusions depend on an arbitrary one-dimensional ordering.

### Architecture selection and adaptation

The selection of architectural models should be evaluated in the context of their relationship to representation and downstream tasks, rather than being viewed as an independent source of methodological innovation. Encoder-only, decoder-only, and linear-attention architectures each offer distinct trade-offs concerning contextual integration, generative capabilities, and computational scalability.

#### Encoder-only architectures (BERT-like): core adaptation for classification and dimensionality reduction

Encoder-only architectures employ stacked Transformer encoder layers and self-attention to model bidirectional context. In the context of ST, these architectures nonlinearly integrate sparse, high-dimensional gene expression matrices with spatial information, effectively mapping the input into a compact latent representation. A notable example is the Spatial Cell Segmentation (SCS) model, which employs eight encoder layers to adaptively aggregate information from a target spot and its 50 neighboring spots [19]. To facilitate precise cell segmentation, the model undertakes two primary tasks: binary classification to ascertain whether a spot is part of a cell, and prediction of the spot’s direction relative to the cell center. By reducing gene expression features to a 64-dimensional hidden space, SCS addresses data sparsity while maintaining biological variability. Consequently, SCS was reported to achieve higher segmentation accuracy and more accurate cell identification than conventional methods on high-resolution Stereo-seq and Seq-scope datasets [19].

#### Decoder-only architectures (GPT-like): efficient implementation for generation and prediction

The decoder-only architecture formulates gene expression prediction as a constrained sequence generation task. In ST applications, spatial information can be integrated into spatially organized image or molecular representations. Subsequently, multi-head self-attention mechanisms amalgamate contextual information across spatially related regions, thereby enabling the capture of long-range spatial dependencies. Concurrently, residual connections aid in the propagation of spatially informed representations through the decoder layers. This architecture can therefore translate histopathology images into molecular profiles while reducing prediction bias caused by morphological heterogeneity. For instance, Magic employs a Transformer decoder to process ViT-B/32-derived image features and predict the expression of 300 genes. Trained on 56 breast cancer ST slices and evaluated on five independent slices, Magic achieved an ~10% higher image–gene expression alignment accuracy than TRIPLEX, the second-best method, based on cosine similarity. In a separate zero-shot transfer experiment, Magic outperformed BLEEP by ~11% when applied to colorectal cancer samples without fine-tuning [20]. Furthermore, the model’s robust zero-shot generalization capability facilitates its application to colorectal cancer and The Cancer Genome Atlas (TCGA) samples without necessitating fine-tuning. These findings highlight the potential of decoder-only models as a promising strategy for addressing cross-cancer heterogeneity and annotation scarcity in ST research.

#### Linear attention and state-space models for ultra-long sequences

Kernelized linear-attention methods and SSMs, such as Mamba, S4, and their variants, provide alternatives to conventional Transformer attention. Kernelized linear-attention methods approximate or reformulate the Softmax attention operation, whereas SSMs model sequence dependencies through recurrent state-space dynamics [21]. In ST, “ultra-long sequences” refer to the large numbers of spatial units, such as spots or cells, that must be jointly modeled in high-density or whole-slide datasets. These approaches can reduce the computational and memory burden associated with quadratic pairwise attention, enabling long-range dependency modeling at lower cost. They also alleviate the compute and memory bottlenecks, including GPU memory overflow, that arise in large-scale ST analyses of whole slides [22]. This scalability allows models to incorporate broader tissue contexts and thereby facilitate the identification of spatial organization and cell-type patterns across large tissue regions. For instance, SANNO uses a kernelized Gumbel-Softmax operator to linearize Graph Transformer attention. Coupled with random feature mapping and approximate sampling, it significantly boosts throughput while preserving complex spatial topologies. Across six biologically independent intra-dataset pairs, SANNO achieved an average overall annotation accuracy of 79.3%, outperforming STELLAR by 15.1%. Across five biologically independent cross-dataset pairs involving different tissues, it achieved an average accuracy of 73.2%, exceeding XGBoost by 10.4%. SANNO also identified novel cell types, achieving up to 99.3% accuracy in the reported intra-dataset evaluation [23].

The computational benefits of kernelized linear-attention methods and SSMs are particularly pertinent to large-scale, high-density, or whole-slide ST datasets. In contrast, Transformer-based models may be more suitable when there is a need for flexible pairwise contextual modeling or multimodal attention. Consequently, the assessment of architectural efficiency should be conducted in conjunction with considerations of spatial resolution, sequence length, and modeling objectives, rather than being regarded as a universal advantage. On the other hand, diffusion-based models may provide benefits for generative reconstruction and uncertainty modeling, albeit typically at the cost of increased computational requirements.

### Pretraining objective: masked gene modeling

Within foundation-model approaches for ST, pretraining objectives define how models learn representations from large unlabeled datasets. Pretraining enables models to learn generalizable molecular and spatial patterns before downstream adaptation, reducing dependence on task-specific labeled data; e.g. Nicheformer demonstrated transferability across spatial composition and spatial label prediction tasks [14]. In ST, masked gene modeling (MGM) is one commonly used objective. MGM masks selected genes or spatial measurements and trains the model to reconstruct them from the remaining molecular and spatial context. This objective primarily supports reconstruction and denoising of incomplete expression profiles. For instance, SpaCKLE combines MGM with spatial geometric constraints to improve the reconstruction of missing gene expression data [24].

The value of MGM can extend beyond simple imputation. Its training signal may support downstream analyses of spatial organization and cellular interactions. Accurate reconstruction requires joint modeling of gene co-expression, spatial organization, and functional links between cells. The learned representations can therefore support robust spatial domain identification. They can also resolve fine-grained tissue structures not covered by reference atlases [25]. In addition, they can be used to infer cell–cell interaction networks in disease settings [26]. These results suggest that, in foundation models for spatiotemporal omics, the role of MGM extends beyond handling missing data. The mask-and-reconstruct objective has the potential to capture statistical dependencies among genes and spatial units; however, it is crucial to recognize that these dependencies should not be construed as regulatory or causal relationships without validation through perturbational or experimental methods. Thus, MGM should be interpreted primarily as a self-supervised reconstruction objective, while stronger claims about regulatory mechanisms require independent biological or perturbational validation. Table 1 summarizes representative AI, generative, and foundation-model applications in ST, covering their tasks, prediction targets, input modalities, evaluation metrics, validation levels, and representative methods for analyzing spatial molecular organization.

**Table 1:** Task-level evidence summary of representative AI, generative-modeling, and foundation-model applications in ST.

Task category	Prediction target/biological question	Input modalities	Dataset/cohort	Reference standard	Metric	Leakage risk	Validation level	Split/transfer setting	Representative methods
Spatial domain identification	Identification of spatial domains, tissue regions, cellular states, niches, and tissue organization patterns	Spatial gene expression profiles and spatial coordinates; optionally H&E images, cell-type annotations, or additional omics	Public ST datasets from brain, tumor, and other tissues; single-section, multi-section, cross-platform, and simulated settings	Expert or histological annotations, anatomical regions, curated domain labels, or reproducible marker-defined patterns	ARI, NMI, F1-score, silhouette score, spatial autocorrelation, and marker-enrichment analysis	Random spot-level splitting; adjacent spots from the same section appearing in both training and test sets; labels derived from input expression; patient-, donor-, or slide-level confounding	Level 1: mainly retrospective computational validation, with limited independent donor, tissue, or platform validation	Section- or sample-level split; cross-tissue or cross-platform evaluation	STAMP [55]; MCGAE [27]; SAMGCN [39]; spaLLM [38]; SpaNCMG [17]; BMCST [22]
Expression reconstruction	Imputation of missing genes, prediction of spatial expression, resolution enhancement, denoising, and reconstruction of tissue molecular features	ST expression, spatial coordinates, H&E images, and/or scRNA-seq reference profiles	Matched histology–ST datasets, paired or adjacent-section datasets, high-resolution spatial platforms, and masked gene or simulated settings	Held-out measured genes, matched high-resolution measurements, imaging-based transcriptomics, or experimentally measured expression	Pearson/Spearman correlation, RMSE, MAE, R^2^, gene-wise recovery, spot-wise recovery, and structural similarity	Information leakage through gene selection or masking; overlap of patients, slides, serial sections, or image tiles; use of same specimen reference profiles in both training and testing	Level 1–2: retrospective paired-data validation, with some cross-slide or cross-platform evaluation	Masked gene or held-out-expression evaluation; cross-slide or cross-platform evaluation	FmH2ST [8]; XFuse [40]; U-FISH [50]; BrST-Net [60]; TCGN [61]; SpatialScope [62]
Perturbation-response prediction	Prediction of transcriptional or cellular-state changes caused by genetic, pharmacological, or microenvironmental perturbations	Baseline expression profiles, perturbation or treatment labels, spatial information, and optionally histology, pathways, or biological knowledge	Perturb-seq, drug-perturbation, pre-/post-treatment, and disease-specific spatial datasets; *in silico* perturbation settings	Observed post-perturbation profiles, differential-expression signatures, or experimentally measured response phenotypes	Pearson/Spearman correlation, RMSE, MAE, differential-expression recovery, AUROC/AUPRC, F1-score, and calibration	Patient, drug, cell-line, perturbation, or treatment overlap between training and test sets; use of post-treatment information during feature construction; pathway or database leakage	Level 1: mainly retrospective or *in silico* validation; independent experimental and prospective validation remains limited	Patient-, drug-, cell-line-, perturbation-, or treatment-level split; evaluation on unseen perturbations or biological conditions	SpaRx [63] and other perturbation-aware generative or foundation-model approaches discussed in the manuscript
Cell–cell interaction	Inference of ligand–receptor communication, spatially organized cellular interactions, relay networks, and tissue-regulatory relationships	ST or scRNA-seq expression, spatial coordinates, cell-type annotations or deconvolution results; optionally histology, protein data, and prior interaction knowledge	Visium, Slide-seq, MERFISH/seqFISH, and paired single-cell/spatial datasets from normal and diseased tissues	Experimentally supported interactions, spatial proximity, matched protein or imaging evidence, and curated ligand–receptor resources	AUROC/AUPRC, precision, recall, F1-score, interaction-enrichment scores, proximity statistics, and cross-cohort reproducibility	Circular evaluation against the same interaction database used as model input; pseudo-label leakage from cell-type annotations; neighboring cells or spots split across datasets; tissue-specific prior information	Level 1, exploratory: mainly computational benchmarking and biological-plausibility analysis, with limited orthogonal experimental confirmation	Spatially held-out evaluation with independent tissue- or cohort-level testing	CellNEST [46]; spaCl [34]; CELLetter [64]
Cross-modal alignment	Alignment of spatial expression, cell identity, histology, other omics, and biological knowledge for label transfer, representation learning, multimodal integration, and regulatory interpretation	scRNA-seq, ST, spatial coordinates, H&E images, protein or epigenomic modalities, and text or prior biological knowledge	Matched or partially paired scRNA-seq–ST datasets, multi-platform spatial datasets, and multimodal spatial-omics cohorts	Known cell-type labels, shared anchors, matched serial sections, paired multi-omics measurements, or expert annotations	Cell-type transfer accuracy, macro-F1, ARI/NMI, embedding-alignment scores, batch-mixing scores, gene expression correlation, and cross-modal retrieval accuracy	The same specimen, donor, section, image patch, or cell type appearing across training and test sets; anchor selection using all samples; shared labels or reference atlases across splits	Level 1–2: retrospective alignment and label transfer evaluation, with limited held-out donor or platform testing	Matched or partially paired multimodal data; held-out donor-, section-, or platform-level transfer	SELF-Former [6]; CellMemory [13]; SANNO [23]; Nicheformer [14]; CELLama [12]
Cell segmentation	Segmentation of individual cells and delineation of cell boundaries in ST data for cell-level transcript assignment and downstream spatial analysis	Spatial transcript coordinates and gene identities; optionally nuclei or histology images and initial nuclear-segmentation results	Sequencing-based subcellular ST and imaging-based ST datasets from multiple tissues and experimental platforms; simulated and annotated spatial datasets	Manually curated cell-instance annotations only for seqFISH+; indirect expression correlation benchmark; simulated ground-truth assignments	Intersection over Union, precision, recall, F1-score, segmentation accuracy, transcript assignment accuracy, Pearson correlation for expression benchmark	Overlap of tiles, fields of view or specimens across train/test splits; adjacent cells across splits; reused nuclear/preprocessing labels; in-section training-prediction with training tiles sampled from the same section	Level 1–2: retrospective computational validation across independent external datasets and technology platforms	No sample-level train-test split within each dataset; model trains and infers on the same section; evaluated on fully external cross-tissue and cross-platform datasets	SCS [19]

Validation levels were defined as follows:

Level 1 indicates mainly retrospective computational or *in silico* validation based on held-out data, cross-validation, simulated settings, or task-specific quantitative metrics, with limited or no independent biological, experimental, donor-level, or prospective confirmation.

Level 1–2 indicates computational evaluation supplemented by partial external, orthogonal, cross-slide, cross-platform, cross-donor, or biologically informed evidence, but without comprehensive independent biological or experimental validation.

These levels describe the strength of the reported validation evidence rather than the overall quality, novelty, or biological importance of a method. Within each task category, the datasets/cohorts, reference standards, metrics, leakage risks, validation levels, and split/transfer settings summarize the typical evaluation practice associated with the representative methods listed in the last column; method-specific details are described in the main text.

## Task-driven biological discovery and case studies

### Precise spatial domain identification

Spatial domain identification is essential for dissecting heterogeneity in tissue microenvironments. However, conventional clustering methods rely mainly on transcriptional similarity. They often do not model spatial relationships explicitly. As a result, they may fail to separate tissue regions that contain similar cell types but differ in functional state. This limitation is especially clear in the dorsolateral prefrontal cortex, where layer 3 and layer 4 have similar expression profiles and are difficult to distinguish [27]. In contrast, context-aware models can integrate local neighborhood structure with broader spatial information. Spatial coordinates therefore provide an important constraint for domain assignment rather than an auxiliary feature.

Under this view, a spatial domain is modeled as a structural unit defined jointly by expression patterns, neighborhood configuration, and tissue location. This design enables the model to infer boundaries that are more biologically meaningful. For instance, SELF-Former can recover spatial expression patterns of boundary-associated genes, including v-erb-b2 erythroblastic leukemia viral oncogene homolog (*ERBB*), cadherin 1 (*CDH1*), and marker of proliferation Ki-67 (*MKI67*). It can therefore resolve the continuous transition between tumor microenvironments with high versus low expression [6]. In the human breast cancer (HBC) human breast cancer dataset (CID3586), generated using 10× Visium, the context-aware boundary between the tumor core and tumor edge agreed well with tissue morphology and immunohistochemistry annotations. Using structural similarity as the evaluation metric, the proposed method achieved a score of 0.895, compared with 0.717 for SpaDiT under the same evaluation setting [28]. These results suggest that context-aware models can improve spatial domain delineation when their predictions are supported by independent morphological or molecular evidence.

### *In silico* perturbation and cross-modal alignment

*In silico* perturbation should be distinguished from masked gene reconstruction and causal intervention. MGM is primarily a reconstruction task rather than a simulation of gene knockout, whereas *in silico* perturbation predicts hypothetical changes associated with altering a target gene. Unless supported by genuine intervention data, such predictions should be interpreted as hypothesis-generating rather than causal. For example, computational perturbation studies have predicted that collagen type XI alpha 1 chain (COL11A1) silencing may be associated with extracellular matrix remodeling and altered immune infiltration in ovarian cancer [29], while aldolase A (ALDOA) silencing may reduce hypoxic areas and weaken angiogenic gradients in breast cancer [30]. Virtual perturbation of C-C motif chemokine ligand 20 (*CCL20*) has also been reported to predict reduced Treg recruitment and changes in cluster of differentiation 8 (CD8^+^) T cells localization in prostate cancer [31]. These results illustrate the potential of *in silico* perturbation for prioritizing candidate regulators and hypotheses, but the predicted effects should not be interpreted as experimentally established causal responses.

This predictive ability depends on how accurately tissue structure and molecular states are represented. Cross-modal alignment offers a methodological approach for synthesizing complementary data derived from histological analysis, spatial coordinates, and transcriptomic profiles. Histological analysis elucidates tissue architecture and delineates boundaries, while transcriptomic profiles provide insights into cellular states and functional activities. By projecting these modalities into a shared representation, models can learn correspondences between morphology and molecular signals [32]. Such integration may improve spatial resolution and help identify molecular states that are difficult to distinguish from transcriptomic information alone. For example, multimodal joint can refine low-resolution spots into finer spatial patterns while preserving region-specific marker gene signals [33]. However, cross-modal alignment may also propagate modality-specific noise or introduce errors when the spatial registration or molecular measurements are inaccurate in modeling. Its value therefore depends on the quality and compatibility of the input modalities.

### Inference of cell–cell interaction networks

In ST analysis, cell–cell communication can be by integrating molecular profiles with spatial relationships rather than relying solely on pairwise expression correlations. Within this framework, attention-based and graph-based models can incorporate ligand–receptor information together with spatial neighborhoods to model and characterize potential communication networks. Attention weights may help prioritize relationships between neighboring or spatially separated cells, while end-to-end graph representation learning can encode interaction-related features without relying exclusively on predefined ligand–receptor pairs [34, 35]. These approaches may help distinguish candidate interactions from associations driven primarily by physical proximity and identify potential long-range communication patterns. However, model-derived interaction scores or attention weights should be interpreted as computational evidence rather than direct measurements of cellular communication. Experimental or orthogonal validation is required to establish the underlying signaling mechanism. Figure 1 summarizes the conceptual pipeline, in which multimodal ST inputs are represented as spatial units and processed by Transformer- or state-space-based architectures. The resulting representations can be applied to downstream tasks including spatial domain identification, *in silico* perturbation, expression reconstruction, and interaction inference.

## Challenges: benchmarking, hallucination, and scaling laws

Despite the growing capabilities of foundation models in ST, their biological reliability depends critically on how model outputs are generated and evaluated. Two particularly important challenges are hallucination and data leakage. Hallucination can result in the generation of biologically plausible yet unsubstantiated molecular or spatial patterns, particularly when generative models are employed to reconstruct sparse measurements. Conversely, data leakage can cause an overestimation of performance metrics when spatially correlated regions from the same tissue section are inadvertently included in both training and test datasets. These challenges are especially significant in ST due to the inherent spatial dependence, measurement sparsity, and biological heterogeneity, which complicate the differentiation between computational artifacts and authentic biological signals. Therefore, rigorous validation strategies are essential for assessing the reliability and generalizability of foundation models in spatial biological analysis.

### Hallucination

#### Is generated gene expression a biological finding or statistical noise?

ST models may produce “hallucinated” signals when they integrate multi-view data or use foundation-model approaches to impute sparse expression profiles. These signals can become disconnected from true biological mechanisms. This problem is especially evident in contrastive learning and expression imputation tasks. Contrastive learning usually constructs multi-view data through random perturbations [36]. However, if biological constraints are weak or absent, these perturbations may disrupt the original expression semantics and spatial context. As a result, the model may learn false statistical associations rather than real features of the microenvironment [37]. For example, spatially correlated cell states or technical signals within a microenvironment may be incorrectly associated with a biological condition, resulting in spurious statistical associations. A similar problem appears when foundation-model approaches are used to complete sparse ST expression data. Because spatial transcriptomic measurements can contain sparse or limited gene expression information, generated signals may reflect both biological heterogeneity and measurement-related effects and therefore require independent validation before being interpreted as biological discoveries [38]. Figure 2 illustrates a four-step foundation-model-based workflow for mitigating AI hallucinations in ST: generative prediction, confidence-based filtering and reference retrieval, experimental validation, and iterative model refinement.

**Figure 2:**
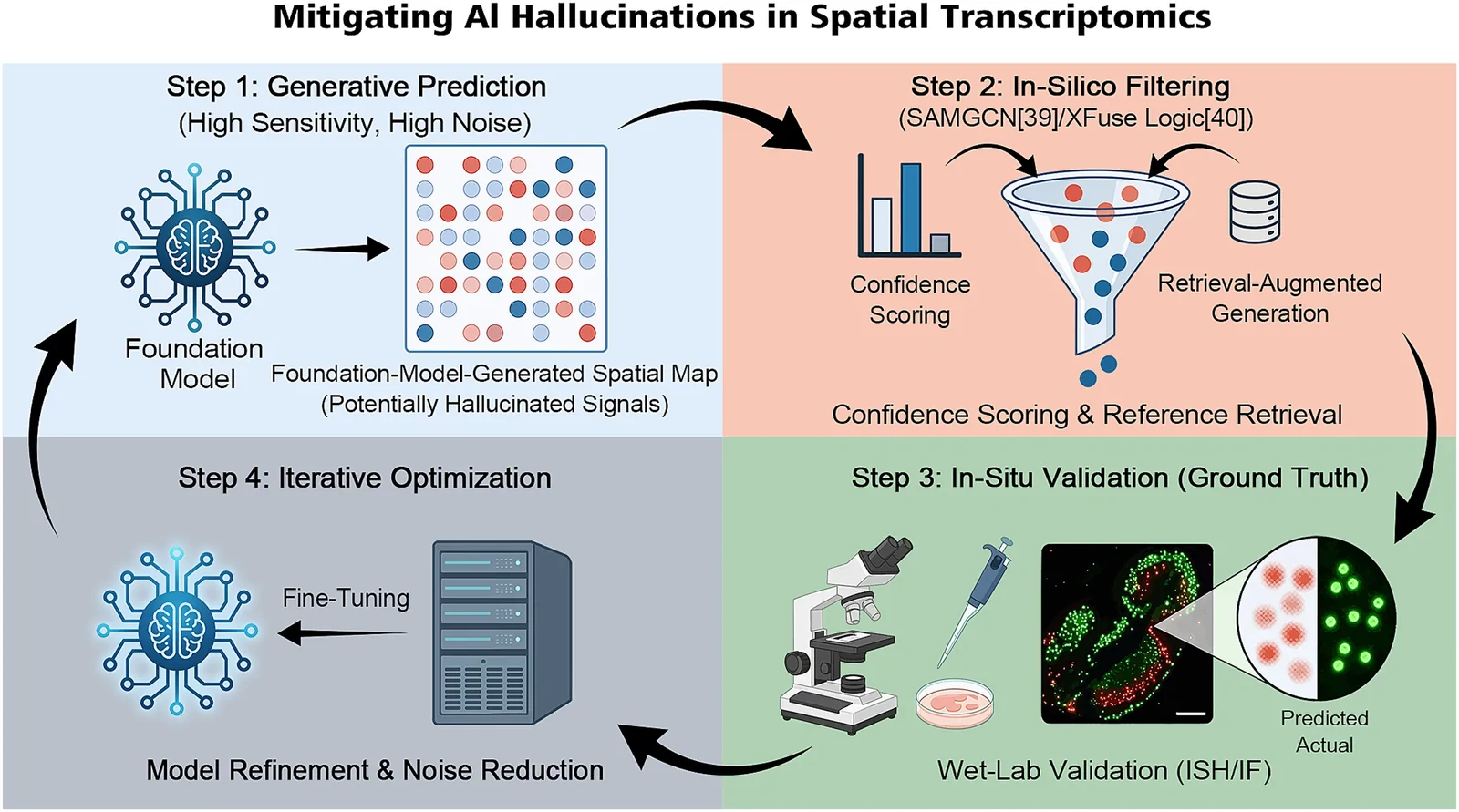
A four-step foundation-model-based workflow for mitigating AI hallucinations in ST. Step 1: generative prediction produces a candidate spatial map with high sensitivity but potential hallucinated signals. Step 2: *in silico* filtering combines confidence scoring (e.g. SAMGCN [39]) with retrieval-augmented generation against reference datasets (e.g. XFuse logic [40]). Step 3: *in situ* validation compares predicted and measured signals using wet-lab assays such as *in situ* hybridization or immunofluorescence. Step 4: iterative optimization feeds validation results back into fine-tuning for model refinement and noise reduction.

#### Solutions: confidence scoring, retrieval-augmented generation, and wet-lab validation

To reduce hallucination, the reliability of model outputs must be improved. A practical solution is to combine confidence scoring with retrieval-augmented generation. This strategy can be further strengthened by adding a wet-lab-driven validation loop. SAMGCN is a representative example. It allows model outputs to be ranked by confidence, so unreliable predictions can be filtered out before downstream analysis [39]. Deep generative models provide a complementary solution. By integrating multiple data types and retrieving relevant reference datasets, they can reduce unsupported signal generation. XFuse shows this idea clearly. It combines multimodal information and uses external evidence, such as scRNA-seq data, to constrain the generated expression distribution. As a result, expression hallucinations that deviate from true tissue states can be reduced [40].

The integration of a wet-lab-driven loop significantly enhances this framework. Central to this process is a closed cycle comprising model generation, experimental validation, and model refinement. Initially, candidate gene expression patterns are identified through confidence scoring and retrieval-based verification, forming the basis for targeted experimental hypotheses. Subsequently, wet-lab techniques, such as *in situ* hybridization and immunofluorescence, are employed to ascertain the authenticity of these patterns, thereby distinguishing genuine biological signals from statistical noise. Evidence from expansion sequencing underscores the efficacy of this approach. When combined with antibody staining, it enables the mapping of RNA signals to cellular and subcellular structures, facilitating the detection of expression patterns influenced by tissue context. For instance, the expression of S100 calcium-binding protein A8 (*S100A8*) was found to be ~four-fold higher in immunoglobulin heavy constant gamma 1 (*IGHG1*)-positive B cells in proximity to epidermal growth factor receptor (*EGFR*)-positive tumor cells. This spatially restricted pattern is more consistent with local tumor-immune interaction than with random statistical fluctuation [41].

Evidence from multi-platform ST studies of formalin-fixed paraffin-embedded (FFPE) tumor samples also shows the need for experimental validation. If computational results are used alone, cell segmentation errors, background staining, or probe noise may be mistaken for abnormal co-expression. More reliable interpretation can be achieved when hematoxylin and eosin (H&E), multiplex immunofluorescence, and expert pathology review are considered together. This helps distinguish false co-expression from biologically meaningful expression patterns. For instance, co-expression of cluster of differentiation 4 (CD4) and cluster of differentiation 8 alpha (*CD8A*) in some T cells should not be treated automatically as a technical artifact. In tumors and other pathological conditions, it may instead represent a specific immune phenotype with biological relevance [42]. Finally, validated data can be fed back into the model for parameter refinement. This can reduce the risk of later hallucination and improve the biological reliability of generated results. Overall, this strategy provides a practical framework for separating model-generated signals from independently supported biological evidence.

### Data leakage and generalization ability

#### Data leakage controversy in train/test splits across different regions of the same tissue section

One core feature of ST data is spatial correlation. Gene expression patterns in different regions of the same tissue section are naturally continuous and related. Therefore, splitting different regions from the same tissue section into training and test sets may introduce data leakage. Spatially contiguous regions may exhibit analogous gene regulatory networks, cell-type compositions, and local microenvironmental signals. When these regions are partitioned between training and test datasets, the model might leverage these shared spatial patterns instead of learning features that generalize to independent tissues or samples. Consequently, the resulting performance metrics may be unduly optimistic. To reduce this risk, GeneLink+ used a hard negative sampling strategy. This ensured that negative samples did not overlap between the training and test sets. The ratio of positive to negative samples was also adjusted according to network density, reducing potential leakage effects [43].

A similar concern was addressed in STPath during pretraining. Samples from downstream tasks were strictly excluded, and a zero-shot evaluation setting was adopted. This design reduced the risk that spatial information from the same tissue section would be indirectly incorporated into the training process and made evaluation more reflective of independent generalization [44]. These examples highlight the need for sample-level or tissue-level partitioning, rather than relying only on spatially separated regions from the same section.

#### Evaluation of zero-shot transfer across species and organs

The practical value of ST tools depends on their ability to generalize across species and organs. Zero-shot transfer evaluation tests this ability by applying a model to a new species, organ, or dataset without task-specific fine-tuning on the target data. PAST showed stable performance by integrating reference data from multiple sources. Its performance remained robust across four sequencing platforms, four species (human, mouse, salamander, and zebrafish), and seven tissue types. For example, mouse cortical scRNA-seq data could be used as a reference to analyze ST data from the mouse primary visual cortex. The method was also extended successfully to spatial domain identification in regenerating salamander brain tissue and developing zebrafish embryonic tissue. These results support the effectiveness of cross-species transfer [45].

CellNEST provides another example. It achieved accurate detection of cell–cell communication in datasets from human lymph node, lung cancer, colorectal cancer, pancreatic cancer, and mouse hypothalamus. No fine-tuning was required for a specific species or organ. This method uses a graph attention network and contrastive learning to capture general spatial communication patterns. As a result, key signaling pathways could be identified in both human tumors and mouse neural tissue. Examples include the fibronectin 1 (*FN1*)-ribosomal protein SA (*RPSA*) pathway in human pancreatic cancer and the oxytocin (*Oxt*)-oxytocin receptor (*Oxtr*) pathway in the mouse hypothalamus. These findings indicate strong zero-shot transfer ability across species and organs [46]. Taken together, the performance of these two types of tools suggests that these methods can move beyond species and organ boundaries when spatial information and multimodal features are effectively integrated. This provides a more general solution for ST analysis in complex biological settings.

Related foundation-model studies outside ST further highlight the importance of task- and data-dependent evaluation. scDrugMap benchmarked multiple single-cell foundation models for drug-response prediction and showed substantial variation in performance across datasets and biological contexts [47]. PhenoProfiler similarly demonstrated the value of robust evaluation across independent plates, datasets, and out-of-distribution conditions for image-based phenotypic representation learning [48]. Together, these studies suggest that the advantages of different architectures should not be assessed solely by predictive accuracy, but also by their computational efficiency, scalability, multimodal compatibility, and ability to generalize across biological and technical settings. For ST, these considerations are particularly important because spatial platforms differ substantially in resolution, sequence length, molecular coverage, and available imaging information.

### Does the scaling law hold in biology?

Current evidence from biological foundation-model studies suggests that performance gains from increasing training data may be nonlinear and subject to diminishing returns. Instead, performance often shows nonlinearity, threshold effects, and diminishing returns. Results from the SEQUOIA model in tumor gene expression prediction support this view [49]. Model performance was positively associated with training sample size, but the relationship was not linear. In breast invasive carcinoma (BRCA), performance continued to decline when the training set was downsampled to 20%–40%. However, once the sample size reached a certain threshold, the benefit of adding more data became much smaller. At the same time, in BRCA, where samples were relatively abundant, the performance gain of a linearized Transformer over a standard Transformer was only about 10%. In pancreatic adenocarcinoma, where samples were limited, the gain reached 160%. These results suggest that the benefit of model scaling or structural optimization is constrained by both data size and task difficulty.

Similar patterns have been reported in other studies. The U-FISH model showed that good performance could already be achieved with about 300 training images, with an F1 score above 0.85. When the number of training images increased to 1800, the F1 score exceeded 0.9. After that point, however, further increases in data led to only very limited improvement [50]. StereoMM showed a related result. When different hidden-layer node settings were tested, no significant statistical differences were found in Adjusted Rand Index or Normalized Mutual Information across model sizes. This suggests that simply increasing model complexity did not produce stable performance gains [51]. Taken together, these findings indicate that model improvement in biological tasks follows a limited scaling pattern. Early gains can be clear, but performance often reaches saturation later.

A main reason for this pattern is the nature of biological data. Such data usually contain high noise, strong heterogeneity, and limited effective information. ST data are influenced by technical variation, including staining differences, imaging quality, sequencing depth, and batch effects [52]. They are also affected by biological factors, such as intratumor heterogeneity, continuous transitions between cell states, and differences among individual patients [53]. As a result, models must identify relatively limited stable signals within a complex background. Because of this, an increase in sample number does not necessarily mean a similar increase in useful information. This is especially true when new data come mainly from similar samples or the same experimental conditions. In such cases, the contribution to model generalization may be small.

Another challenge is that spatial domain boundaries are often not sharply discrete. Instead, they tend to show gradual transitions. This makes the labels themselves partly ambiguous and somewhat subjective [54]. In addition, distribution shifts across platforms, experimental workflows, and cohorts make it difficult for scaling benefits observed in one dataset to transfer reliably to others. Therefore, scaling laws are not entirely absent in biology, but they are strongly condition-dependent. In lower-level tasks, such as expression prediction and image reconstruction, larger datasets and larger models may still improve performance. In more complex tasks, such as spatial domain identification, mechanism analysis, and clinical stratification, performance depends more on data quality, label reliability, and biological priors. Overall, optimization of biological models is more likely to depend on high-quality data, domain knowledge, and multimodal integration than on scale expansion alone. Table 2 summarizes key challenges in applying foundation and generative models to ST, including hallucination, data leakage, and failed scaling laws, together with their causes, potential solutions, and representative studies.

**Table 2:** Summary of key challenges, potential solutions, and representative studies for foundation-model approaches in ST.

No.	1		2	3	
Challenge	Hallucination				
Description	Foundation and generative models applied to ST may produce biologically plausible but unsupported molecular or spatial patterns when sparse measurements are reconstructed or multi-view data are integrated without biological constraints.				
Representative studies	SAMGCN. SAMGCN is a representative example: it allows model outputs to be ranked by confidence, so unreliable predictions can be filtered out before downstream analysis.		XFuse. XFuse integrates multimodal information and uses external evidence, such as scRNA-seq data, to constrain the generated expression distribution, reducing expression hallucinations that deviate from true tissue states.	Multi-platform FFPE study. Multi-platform ST studies of FFPE tumor samples show that, if computational results are used alone, cell segmentation errors, background staining, or probe noise may be mistaken for abnormal co-expression.	
Potential solutions	Combine confidence scoring with retrieval-augmented generation; filter low-confidence predictions before downstream analysis.		Deep generative multimodal integration constrained by retrieved reference datasets.	Add a wet-lab-driven validation loop: *in situ* hybridization, multiplex immunofluorescence, H&E and expert pathology review to distinguish genuine signals from statistical noise.	
References	Liu H *et al.* SAMGCN: a spatially-augmented multi-view graph convolutional network for spatial transcriptomics [39]		Bergenstråhle L *et al.* Super-resolved spatial transcriptomics by deep data fusion [40]	Ozirmak Lermi N *et al.* Comparison of imaging-based single-cell resolution spatial transcriptomics platforms [42]	
**No.**	**1**	**2**	**3**	**4**	**5**
Challenge	Data leakage				
Description	Spatially adjacent regions of the same tissue section share gene regulatory networks, cell-type composition, and microenvironmental signals. Splitting such regions across training and test sets can cause data leakage and overly optimistic performance; evaluation should use sample- or tissue-level partitioning and zero-shot transfer settings.				
Representative studies	GeneLink+. GeneLink+ used a hard negative sampling strategy, ensuring that negative samples did not overlap between training and test sets; the positive-to-negative ratio was adjusted according to network density, reducing potential leakage effects.	STPath. During pretraining, STPath strictly excluded samples from downstream tasks and adopted a zero-shot evaluation setting, reducing the risk that spatial information from the same tissue section would be indirectly incorporated into training.	PAST. PAST showed stable zero-shot transfer across four sequencing platforms, four species (human, mouse, salamander, and zebrafish), and seven tissue types without task-specific fine-tuning.	CellNEST. CellNEST achieved accurate cell–cell communication detection in human lymph node, lung cancer, colorectal cancer, pancreatic cancer, and mouse hypothalamus datasets without species- or organ-specific fine-tuning.	scDrugMap/PhenoProfiler. scDrugMap benchmarked multiple single-cell foundation models for drug-response prediction and showed substantial performance variation across datasets and biological contexts; PhenoProfiler demonstrated the value of robust evaluation across independent plates, datasets, and out-of-distribution conditions.
Potential solutions	Hard negative sampling without train/test overlap; sampling ratio adjusted to network density.	Sample-level exclusion of downstream-task samples during pretraining; zero-shot evaluation.	Zero-shot transfer evaluation across species, organs, and platforms.	Graph attention network with contrastive learning to capture general spatial communication patterns; zero-shot cross-species evaluation.	Task- and data-dependent benchmarking across independent datasets and out-of-distribution conditions.
References	Zhang W *et al.* Inferring cell-type-specific gene regulatory network from cellular transcriptomes [43]	Huang TL *et al.* STPath: a generative foundation model for integrating spatial transcriptomics and histopathology [44]	Li Z *et al.* Latent feature extraction with a prior-based self-attention framework for spatial transcriptomics [45]	Zohora FT *et al.* CellNEST reveals cell–cell relay networks using attention mechanisms [46]	Wang Q *et al.* scDrugMap [47]; Li B *et al.* PhenoProfiler [48]
Challenge	Failed scaling laws				
Description	Performance gains from increasing training data in biological foundation models are often nonlinear, with threshold effects and diminishing returns, because biological data contain high noise, strong heterogeneity, and limited effective information.				
Representative studies	SEQUOIA. In tumor gene expression prediction, SEQUOIA’s performance was positively associated with training sample size, but the relationship was not linear: performance declined when the training set was downsampled to 20%–40% and saturated once sample size reached a certain level.		U-FISH. U-FISH achieved an F1 score above 0.85 with about 300 training images and above 0.9 with 1800 images; further increases in data led to only very limited improvement.	StereoMM. Across different hidden-layer node settings, StereoMM showed no significant statistical differences in ARI or NMI, indicating that simply increasing model complexity did not improve performance.	
Potential solutions	Report full data-scaling curves rather than results at a single training set size; identify saturation thresholds.		Determine the minimum sufficient training set size; evaluate diminishing returns explicitly.	Test statistical significance across model sizes before scaling up model complexity.	
References	Pizurica M *et al.* Digital profiling of gene expression from histology images with linearized attention [49]		Xu WZ *et al.* U-FISH: a fluorescent spot detector for imaging-based spatial-omics analysis [50]	Luo BY *et al.* StereoMM: a graph fusion model for integrating spatial transcriptomic data [51]	

**Table 3:** Tokens, sequences, spatial context, and biological interpretations across ST resolutions.

Platform resolution	Token	Sequence or neighborhood	Spatial context	Appropriate interpretation
Spot level	A multicellular capture spot	Ordered or graph-connected neighboring spots	Regional expression, morphology, and mixed cell-type composition	Spatial domains and regional tissue organization
Cellular level	An individual segmented cell	Spatially neighboring cells	Cellular states, local niches, and tissue architecture	Cell states and candidate intercellular interactions
Subcellular level	A transcript, probe signal, or intracellular measurement	Measurements ordered or connected by intracellular coordinates	Subcellular compartment and proximity to cellular boundaries	RNA localization and intracellular organization

## Future perspectives: toward general spatial intelligence

The directions discussed below represent emerging opportunities rather than established capabilities. Their development will require appropriate temporal, multimodal, physical, and clinical validation. Privacy, ethics, and responsible use are addressed as governance considerations and are therefore not included as a separate component of this technical roadmap. These future directions are summarized in Fig. 3.

**Figure 3:**
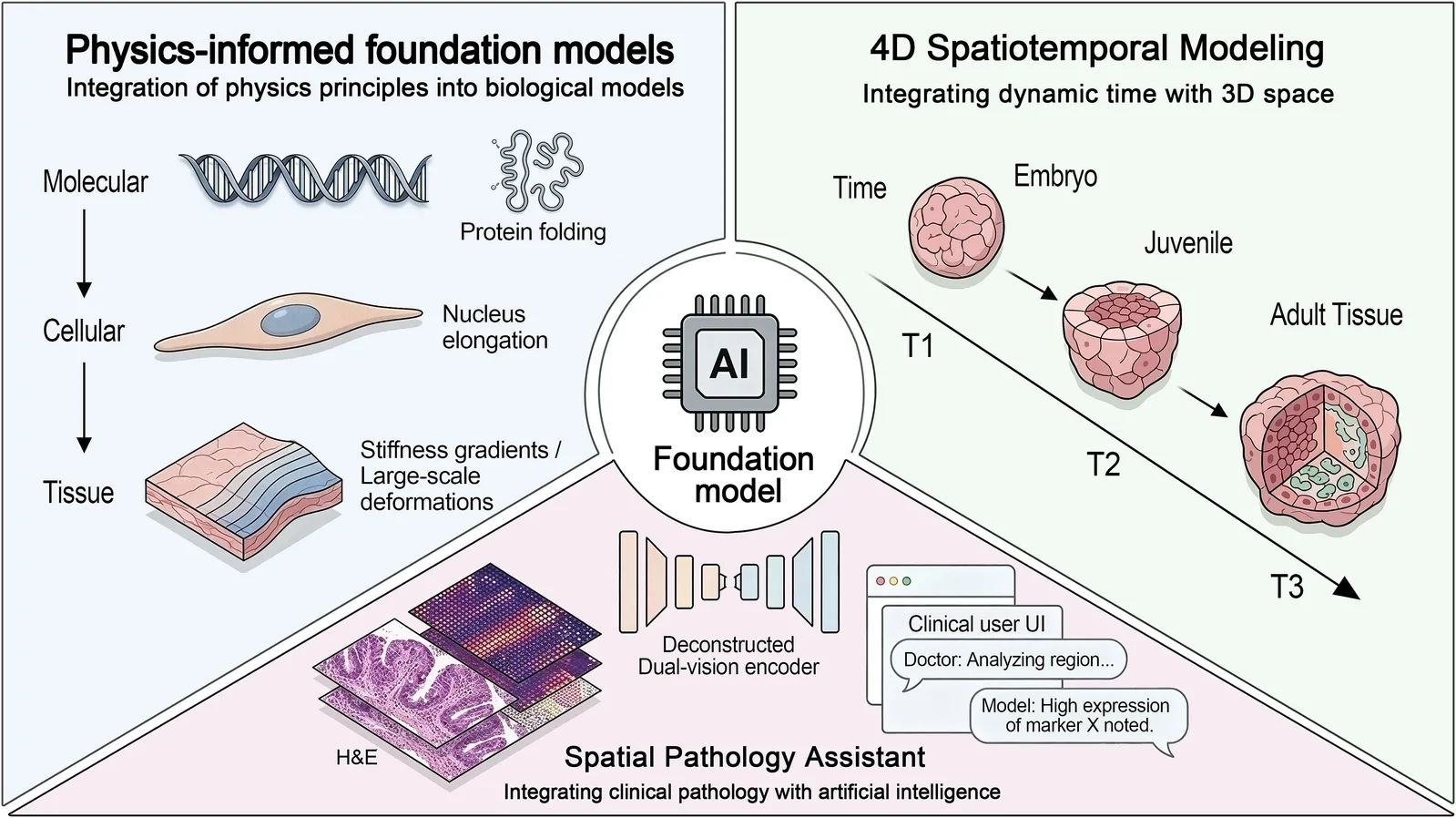
Future perspectives for foundation-model approaches in ST. Three emerging directions are shown: physics-informed foundation models, which integrate physics principles across molecular, cellular, and tissue scales; four-dimensional spatiotemporal modeling, which integrates dynamic time with three-dimensional space across developmental stages; and spatial pathology assistants, which integrate clinical histopathology with foundation-model approaches to support interactive interpretation of spatial molecular data.

### Four-dimensional spatiotemporal modeling

Four-dimensional spatiotemporal modeling includes several tasks with distinct evidentiary requirements. Spatial interpolation aims to estimate unmeasured expressions within observed tissue samples and necessitates validation against withheld spatial measurements. Pseudotemporal ordering is employed to infer biological progression from cross-sectional data and requires corroboration from established developmental stages, lineage markers, or independent trajectory evidence. The registration of serial sections involves aligning adjacent sections or developmental stages and should be assessed using anatomical landmarks or known spatial correspondences. While these tasks reconstruct the organization among existing observations, they do not facilitate prospective temporal prediction. Conversely, longitudinal forecasting endeavors to predict future tissue states, necessitating temporally ordered data and validation against genuinely withheld future observations. Mechanistic dynamical modeling seeks to represent the regulatory, cellular, or physical processes driving temporal change and requires time-resolved, preferably perturbational, data, along with experimental validation.

Existing methods primarily support reconstruction and trajectory inference. STAMP uses Gaussian-process priors to model temporal changes in gene modules in mouse fetal liver [55], whereas Stereopy integrates spatial and temporal features to reconstruct developmental trajectories in mouse embryos from E9.5 to E16.5 [56]. Nevertheless, trajectories derived from developmental stages or aligned cross-sectional samples alone do not constitute a basis for prospective prediction. The capability of foundation models to accurately forecast future tissue states remains an unresolved issue and necessitates evaluation through the use of genuinely withheld temporal observations.

### Spatial pathology assistant

Spatial pathology assistants represent a potential application of vision foundation models and ST. Existing approaches integrate histological features with spatial gene expression and allow natural-language interaction with tissue-level information [57]. By combining morphology, spatial location, and molecular profiles, such systems may support interactive exploration of lesion regions, cellular states, and spatial gene expression patterns. However, their current role is primarily analytical rather than clinical decision-making. Translation to precision diagnosis or treatment will require high-quality paired spatial pathology-transcriptomics datasets, rigorous clinical validation, and reliable deployment in clinical workflows.

### Physics-informed foundation models

Physics-informed foundation models may provide a future direction for incorporating biological and physical constraints into ST modeling. Existing multi-scale frameworks such as MS-CoTF provide a conceptual basis for integrating information across molecular, cellular, and tissue levels [58]. Future models may benefit from the integration of constraints pertaining to molecular diffusion, cellular mechanics, and tissue architecture in conjunction with established biological objectives. A potential strategy involves the concurrent definition of physical and biological characteristics, their integration via cross-scale representations, and the incorporation of physical consistency terms during model optimization. These methodologies could facilitate the differentiation of spatial associations from relationships governed by established physical processes. Nonetheless, the extent to which these constraints enhance biological predictive accuracy or mechanistic interpretation requires empirical validation.

### Privacy and ethics

Model interpretability is essential for the clinical application of foundation-model approaches in ST, as black-box models struggle to gain clinician trust. Approaches like spaLLM enhance transparency through attention weight visualization and linking outputs to biological features. Consequently, interpretability must become a core evaluation criterion for clinical foundation models [38].

Furthermore, given the high sensitivity of ST data [59], robust regulatory frameworks must enforce ethical reviews and informed consent. Privacy should be prioritized through local deployment, de-identification, federated learning, and differential privacy to prevent re-identification risks.

## Conclusion

ST offers a spatial framework for understanding molecular states within tissues, whereas foundation models provide a comprehensive approach for integrating molecular, spatial, and imaging data. This review systematically categorizes existing methodologies, ranging from tokenization and spatial representation to model architectures and subsequent applications. These applications encompass spatial domain identification, expression reconstruction, multimodal alignment, and the inference of potential cell–cell interactions. However, current evidence remains insufficient to equate computational predictions with biological mechanisms. Spatial serialization has the potential to create artificial relationships, while reconstruction processes may produce spurious signals. Additionally, spatially correlated data can lead to leakage and overestimated generalization. In a similar vein, attention patterns and *in silico* perturbations should be considered as computational evidence or testable hypotheses unless corroborated by independent biological validation. Future advancements in this field will necessitate rigorous benchmarking, the use of multimodal and longitudinal datasets, cross-tissue evaluation, and experimental validation. With these precautions in place, foundational models may serve as valuable tools for integrating spatial information and generating testable biological hypotheses. Despite these advances, their roles in mechanistic discovery and clinical translation have yet to be fully established.

## Literature search strategy

This scoping review was conducted to map the literature on foundation-model approaches for ST. The Web of Science Core Collection was searched on 30 January 2026, for records published from 1 January 2020 to 30 January 2026. The core search expression was: TS = [(“spatial transcriptom*” OR “spatially resolved transcriptom*” OR “spatial omic*” OR “spatial gene expression”) AND (“foundation model*” OR “large language model*” OR transformer* OR “attention mechanism*” OR “state space model*” OR Mamba OR diffusion OR “vision-language model*” OR “multimodal model*”)].We included English-language, peer-reviewed studies that developed, applied, or evaluated foundation-model approaches relevant to ST and addressed tokenization, spatial representation, model architecture, generative learning, or at least one downstream biological task covered in this review. Studies without a direct connection to ST, duplicate records, conference abstracts, editorials, non-English publications, and articles lacking sufficient methodological information were excluded. Of 120 records identified, 18 duplicates were removed, leaving 102 records for title and abstract screening. After excluding 27 records, 75 full-text articles were assessed for eligibility; 17 were subsequently excluded. Two authors independently screened titles, abstracts, and full texts, with disagreements resolved by the corresponding author, resulting in 58 included studies. Six additional references were subsequently cited as background literature and were not counted among the included studies; together with the 58 included studies, the reference list therefore comprises 64 entries. Studies were organized under the conceptual framework of “cells as words, tissues as sentences” according to three components—tokenization, spatial representation or serialization, and model architecture—and five downstream biological tasks. The literature screening process and the overall organization of this review are illustrated in Fig. 4 modeling.

**Figure 4:**
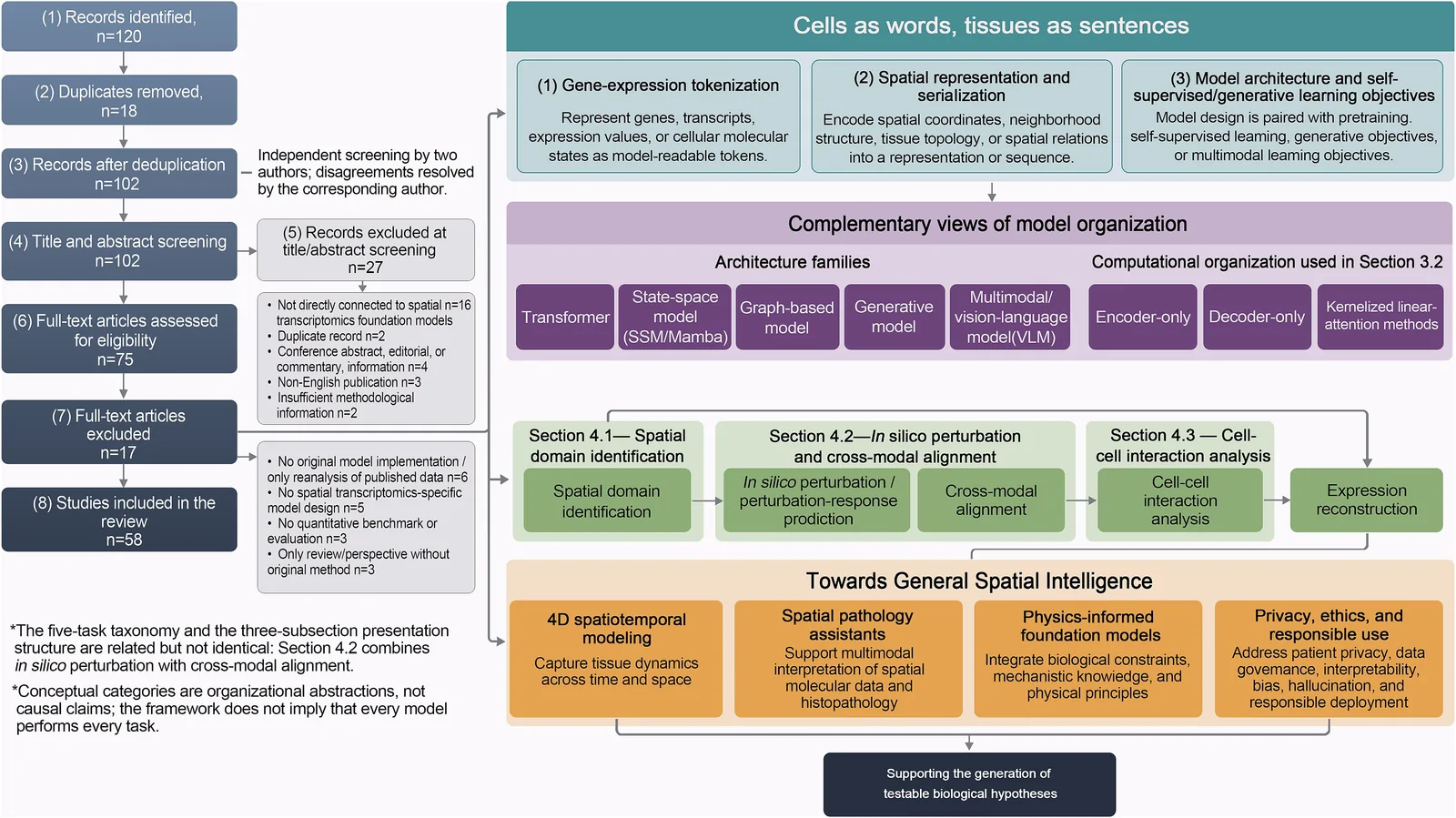
Literature screening process and overall organization of this scoping review. Left: records identified from the Web of Science Core Collection (searched on 30 January 2026) were deduplicated, screened by title and abstract, and assessed in full text, resulting in 58 included studies; six additional references were cited as background literature, yielding 64 references in total. Right: the included studies are organized under the “cells as words, tissues as sentences” framework into three modeling components (gene expression tokenization, spatial representation and serialization, and model architecture and learning objectives), complementary views of model organization, five downstream biological tasks, and future directions toward general spatial intelligence.

## Limitations

Because the search was restricted to the Web of Science Core Collection, relevant studies indexed exclusively in other databases, such as PubMed, Scopus, or IEEE Xplore, may have been missed. In addition, because the search was conducted on 30 January 2026, studies published or indexed after that date were not included.

**Key Points**

- Spatial language representation. Defines cells, spots, or subcellular measurements as spatial tokens and organizes them into context-dependent sequences, providing a common conceptual framework for comparing heterogeneous ST models.
- Generative perspective. Examines how context-aware and generative models extend ST analysis toward reconstruction, prediction, multimodal integration, and candidate biological hypothesis generation, while distinguishing computational evidence from experimentally established mechanisms.
- Critical evaluation. Highlights hallucination, spatial data leakage, limited cross-tissue generalization, and context-dependent scaling, and outlines validation requirements for future spatial foundation models.

## Data Availability

Not applicable.

## References

[1] Theodoris CV, Xiao L, Chopra A et al. Transfer learning enables predictions in network biology. *Nature.* 2023;618:616–24. 10.1038/s41586-023-06139-9 PMC10949956 37258680

[2] Chen WQ, Zhang PZ, Tran TN et al. A visual-omics foundation model to bridge histopathology with spatial transcriptomics. *Nat Methods* 2025;22:1568–82. 10.1038/s41592-025-02707-1 PMC12240810 40442373

[3] Li Y, Lu YJ, Kang C et al. Revealing tissue heterogeneity and spatial dark genes from spatially resolved transcriptomics by multiview graph networks. *Research.* 2023;6:0228. 10.34133/research.0228 PMC10511271 37736108

[4] Bao XY, Bai XS, Liu XP et al. Spatially informed graph transformers for spatially resolved transcriptomics. *Commun Biol* 2025;8:574. 10.1038/s42003-025-08015-w PMC11972348 40188303

[5] Paniagua K, Jin YF, Chen YD et al. Dissection of tumoral niches using spatial transcriptomics and deep learning. *Iscience.* 2025;28:112214. 10.1016/j.isci.2025.112214 PMC11994907 40230519

[6] Chen TY, Wei XD, Xie LX et al. SELF-Former: multi-scale gene filtration transformer for single-cell spatial reconstruction. *Brief Bioinform* 2024;25:bbae523. 10.1093/bib/bbae523 PMC11483138 39413798

[7] Xue SL, Zhu FF, Chen JY et al. Inferring single-cell resolution spatial gene expression via fusing spot-based spatial transcriptomics, location, and histology using GCN. *Brief Bioinform* 2024;26:bbae630. 10.1093/bib/bbae630 PMC11645551 39656774

[8] Wang YQ, Wang J, Xu YY et al. FmH2ST: foundation model-based spatial transcriptomics generation from histological images. *Nucleic Acids Res* 2025;53:gkaf865. 10.1093/nar/gkaf865 PMC12418390 40923764

[9] Yuan JQ, Yu JH, Yi QB et al. SpateCV: cross-modality alignment regularization of cell types improves spatial gene imputation for spatial transcriptomics. *J Transl Med* 2025;23:1188. 10.1186/s12967-025-07245-0 PMC12574271 41162996

[10] Han MH, Yang DK, Cheng JB et al. Towards unified molecule-enhanced pathology image representation learning via integrating spatial transcriptomics. *Pattern Recogn* 2026;172:112458. 10.1016/j.patcog.2025.112458

[11] Shi ZC, Xue SL, Min WW. Geometry-informed multimodal fusion network for enhancing high-density spatial transcriptomics from histology images. *Eng Appl Artif Intell* 2025;162:112647. 10.1016/j.engappai.2025.112647

[12] Park J, Kim S, Kim J et al. CELLama: foundation model for single cell and spatial transcriptomics by cell embedding leveraging language model abilities. *Adv Sci* 2025;12:e2513210. 10.1002/advs.202513210 PMC12866849 41243712

[13] Wang QF, Zhu H, Hu YW et al. CellMemory: hierarchical interpretation of out-of-distribution cells using bottlenecked transformer. *Genome Biol* 2025;26:178. 10.1186/s13059-025-03638-y PMC12183866 40551223

[14] Tejada-Lapuerta A, Schaar AC, Gutgesell R et al. Nicheformer: a foundation model for single-cell and spatial omics. *Nat Methods* 2025;22:2525–38. 10.1038/s41592-025-02814-z PMC12695652 41168487

[15] Hao M, Yan N, Bian H et al. GeST: towards building a generative pretrained transformer for learning cellular spatial context. In: Proceedings of the 20th Machine Learning in Computational Biology Meeting*,* 2025. 10.1101/2025.04.09.648072

[16] Huang WD, Hu YF, Wang LQ et al. Spatially aligned graph transfer learning for characterizing spatial regulatory heterogeneity. *Brief Bioinform* 2025;26:bbaf021. 10.1093/bib/bbaf021 PMC11752617 39841593

[17] Si ZH, Li HS, Shang WJ et al. SpaNCMG: improving spatial domains identification of spatial transcriptomics using neighborhood-complementary mixed-view graph convolutional network. *Brief Bioinform* 2024;25:bbae259. 10.1093/bib/bbae259 PMC11136618 38811360

[18] Wang X, Wang YQ, Wang X et al. Reg2ST: recognizing potential patterns from gene expression for spatial transcriptomics prediction. *Brief Bioinform* 2025;26:bbaf425. 10.1093/bib/bbaf425 PMC12360703 40825238

[19] Chen H, Li DSY, Bar-Joseph Z. SCS: cell segmentation for high-resolution spatial transcriptomics. *Nat Methods* 2023;20:1237. 10.1038/s41592-023-01939-3 37429992

[20] Yan QY, Li X, Cui JN et al. Spatial histology and gene-expression representation and generative learning via online self-distillation contrastive learning. *Brief Bioinform* 2025;26:bbaf317. 10.1093/bib/bbaf317 PMC12229093 40618351

[21] Gu A, Dao T. Mamba: linear-time sequence Modeling with selective state spaces. In: First Conference on Language Modeling, 2024. 10.48550/arXiv.2312.00752

[22] Zhu YR, Zheng X, He X et al. BMCST: balanced multi-view clustering for spatially resolved transcriptomics with Mamba-driven dynamic feature refinement. *Inf Fusion* 2025;124:103425. 10.1016/j.inffus.2025.103425

[23] Zeng YS, Chen YZ, Shangguan NY et al. SANNO: a graph-transformer enhanced optimal transport tool for spatial transcriptomic annotation. *Interdiscip Sci* 2025;17:645–64. 10.1007/s12539-025-00752-0 40789829

[24] Ruiz D, Cárdenas P, Manrique L et al. Completing spatial transcriptomics data for gene expression prediction benchmarking. *Med Image Anal* 2025;106:103754. 10.1016/j.media.2025.103754 40885036

[25] Lee AJ, Dubuc A, Kunst M et al. Data-driven fine-grained region discovery in the mouse brain with transformers. *Nat Commun* 2025;16:8536. 10.1038/s41467-025-64259-4 PMC12504640 41057352

[26] Xiao X, Zhang L, Zhao HY et al. Inferring spatial single-cell-level interactions through interpreting cell state and niche correlations learned by self-supervised graph transformer. *Nat Mach Intell* 2025;7:1188–200. 10.1038/s42256-025-01161-0 PMC13417919 42529556

[27] Yang YW, Zhang CM, Liu ZN et al. MCGAE: unraveling tumor invasion through integrated multimodal spatial transcriptomics. *Brief Bioinform* 2024;26:bbae608. 10.1093/bib/bbae608 PMC11583448 39576225

[28] Sadia RT, Ahamed A, Cheng Q. CausalGenDiff: generative causal diffusion bridges scRNA-seq and spatial transcriptomics. *J Biomed Inform* 2026;173:104966. 10.1016/j.jbi.2025.104966 PMC12739833 41354118

[29] Masatti L, Marchetti M, Pirrotta S et al. The unveiled mosaic of intra-tumor heterogeneity in ovarian cancer through spatial transcriptomic technologies: a systematic review. *Transl Res* 2024;273:104–14. 10.1016/j.trsl.2024.08.001 39111726

[30] Chitra U, Dan S, Krienen F et al. GASTON-Mix: a unified model of spatial gradients and domains using spatial mixture-of-experts. *Bioinformatics.* 2025;41:i523–32. 10.1093/bioinformatics/btaf254 PMC12261403 40662777

[31] Hirz T, Mei S, Sarkar H et al. Dissecting the immune suppressive human prostate tumor microenvironment via integrated single-cell and spatial transcriptomic analyses. *Nat Commun* 2023;14:663. 10.1038/s41467-023-36325-2 PMC9905093 36750562

[32] Wu YL, Xie J, Nie J et al. DANet: spatial gene expression prediction from H&E histology images through dynamic alignment. *Brief Bioinform* 2025;26:bbaf422. 10.1093/bib/bbaf422 PMC12365980 40833275

[33] Li BH, Bao F, Hou YM et al. Tissue characterization at an enhanced resolution across spatial omics platforms with deep generative model. *Nat Commun* 2024;15:6541. 10.1038/s41467-024-50837-5 PMC11297205 39095360

[34] Tang ZY, Zhang TL, Yang BJ et al. spaCl: deciphering spatial cellular communications through adaptive graph model. *Brief Bioinform* 2023;24:10.1093/bib/bbac563. 10.1093/bib/bbac563 PMC9851335 36545790

[35] Li RZ, Yang XR. De novo reconstruction of cell interaction landscapes from single-cell spatial transcriptome data with DeepLinc. *Genome Biol* 2022;23:124. 10.1186/s13059-022-02692-0 PMC9164488 35659722

[36] Liang H, Du X, Zhu B et al. Graph contrastive learning with implicit augmentations. *Neural Netw* 2023;163:156–64. 10.1016/j.neunet.2023.04.001 37054514

[37] Ren S, Liao XY, Liu FR et al. Exploring the latent information in spatial transcriptomics data via multi-view graph convolutional network based on implicit contrastive learning. *Adv Sci* 2025;12:2413545. 10.1002/advs.202413545 PMC12140381 40304359

[38] Li LY, Dong LY, Zhang H et al. spaLLM: enhancing spatial domain analysis in multi-omics data through large language model integration. *Brief Bioinform* 2025;26:bbaf304. 10.1093/bib/bbaf304 PMC12224616 40608008

[39] Liu H, Gao Y, Gao YL et al. SAMGCN: a spatially-augmented multi-view graph convolutional network for identifying spatial domains☆. *Eng Appl Artif Intell* 2025;157:111203. 10.1016/j.engappai.2025.111203

[40] Bergenstråhle L, He B, Bergenstråhle J et al. Super-resolved spatial transcriptomics by deep data fusion. *Nat Biotechnol* 2022;40:476. 10.1038/s41587-021-01075-3 34845373

[41] Alon S, Goodwin DR, Sinha A et al. Expansion sequencing: spatially precise in situ transcriptomics in intact biological systems. *Science.* 2021;371:eaax2656. 10.1126/science.aax2656 PMC7900882 33509999

[42] Ozirmak Lermi N, Molina Ayala M, Hernandez S et al. Comparison of imaging based single-cell resolution spatial transcriptomics profiling platforms using formalin-fixed paraffin-embedded tumor samples. *Nat Commun* 2025;16:8499. 10.1038/s41467-025-63414-1 PMC12474935 41006245

[43] Zhang W, Shao BW, Li WR et al. Inferring cell-type-specific gene regulatory network from cellular transcriptomics data with GeneLink. *Brief Bioinform* 2025;26:bbaf359. 10.1093/bib/bbaf359 PMC12286779 40702704

[44] Huang TL, Liu TY, Babadi M et al. STPath: a generative foundation model for integrating spatial transcriptomics and whole-slide images. *npj Digit Med* 2025;8:659. 10.1038/s41746-025-02020-3 PMC12618518 41238784

[45] Li Z, Chen XY, Zhang XG et al. Latent feature extraction with a prior-based self-attention framework for spatial transcriptomics. *Genome Res* 2023;33:1757–73. 10.1101/gr.277891.123 PMC10691543 37903634

[46] Zohora FT, Paliwal D, Flores-Figueroa E et al. CellNEST reveals cell-cell relay networks using attention mechanisms on spatial transcriptomics. *Nat Methods* 2025;22:1505–19. 10.1038/s41592-025-02721-3 PMC12240806 40481363

[47] Wang Q, Pan Y, Zhou M et al. scDrugMap: benchmarking large foundation models for drug response prediction. *Nat Commun* 2025;17:730. 10.1038/s41467-025-67481-2 PMC12820318 41381537

[48] Li B, Zhang B, Zhang C et al. PhenoProfiler: advancing phenotypic learning for image-based drug discovery. *Nat Commun* 2025;17:793. 10.1038/s41467-025-67479-w PMC12824340 41392045

[49] Pizurica M, Zheng YN, Carrillo-Perez F et al. Digital profiling of gene expression from histology images with linearized attention. *Nat Commun* 2024;15:9886. 10.1038/s41467-024-54182-5 PMC11564640 39543087

[50] Xu WZ, Cai HY, Zhang Q et al. U-FISH: a fluorescent spot detector for imaging-based spatial-omics analysis and AI-assisted FISH diagnosis. *Genome Biol* 2025;26:261. 10.1186/s13059-025-03736-x PMC12400573 40890868

[51] Luo BY, Teng F, Tang G et al. StereoMM: a graph fusion model for integrating spatial transcriptomic data and pathological images. *Brief Bioinform* 2025;26:bbaf210. 10.1093/bib/bbaf210 PMC12100622 40407386

[52] Fang DH, Min WW. SpaCross deciphers spatial structures and corrects batch effects in multi-slice spatially resolved transcriptomics. *Commun Biol* 2025;8:1393. 10.1038/s42003-025-08810-5 PMC12484869 41028333

[53] Li R, Ferdinand JR, Loudon KW et al. Mapping single-cell transcriptomes in the intra-tumoral and associated territories of kidney cancer. *Cancer Cell* 2022;40:1583–1599 e10. 10.1016/j.ccell.2022.11.001 PMC9767677 36423636

[54] Leng J, Yu J, Wu LY et al. Flexible integration of spatial and expression information for precise spot embedding via ZINB-based graph-enhanced autoencoder. *Commun Biol* 2025;8:556. 10.1038/s42003-025-07965-5 PMC11971412 40186054

[55] Zhong CW, Ang KS, Chen JM. Interpretable spatially aware dimension reduction of spatial transcriptomics with STAMP. *Nat Methods* 2024;21:1504–13. 10.1038/s41592-024-02463-8 PMC11541207 39407016

[56] Fang SS, Xu MY, Cao L et al. Stereopy: modeling comparative and spatiotemporal cellular heterogeneity via multi-sample spatial transcriptomics. *Nat Commun* 2025;16:3741. 10.1038/s41467-025-58079-9 PMC12012134 40258830

[57] Li YW, Zhang YC, Wang CY et al. Mini-Gemini: mining the potential of multi-modality vision language models. *IEEE Trans Pattern Anal Mach Intell* 2024;48:3530. 10.1109/TPAMI.2025.3637265 41296951

[58] Song Z, Zhen XC. MS-CoTF: multi-scale chain-of-thought fusion for interpretable biological reasoning with large language models. *Comput Biol Med* 2026;203:111467. 10.1016/j.compbiomed.2026.111467 41570614

[59] Zhong X, Li S, Chen Z et al. Considerations for patient privacy of large language models in health care: scoping review. *J Med Internet Res* 2025;27:e76571. 10.2196/76571 PMC12680930 41269747

[60] Rahaman MM, Millar EKA, Meijering E. Breast cancer histopathology image-based gene expression prediction using spatial transcriptomics data and deep learning. *Sci Rep* 2023;13:13604. 10.1038/s41598-023-40219-0 PMC10442349 37604916

[61] Xiao X, Kong Y, Li RH et al. Transformer with convolution and graph-node co-embedding: an accurate and interpretable vision backbone for predicting gene expressions from local histopathological image. *Med Image Anal* 2024;91:103040. 10.1016/j.media.2023.103040 38007979

[62] Wan XM, Xiao JS, Tam SST et al. Integrating spatial and single-cell transcriptomics data using deep generative models with SpatialScope. *Nat Commun* 2023;14:7848. 10.1038/s41467-023-43629-w PMC10687049 38030617

[63] Tang ZY, Liu X, Li ZT et al. SpaRx: elucidate single-cell spatial heterogeneity of drug responses for personalized treatment. *Brief Bioinform* 2023;24:bbad338. 10.1093/bib/bbad338 PMC10555713 37798249

[64] Wu W, Huang JF, Jiang Y et al. CELLetter: leveraging large language model and dual-stream network to identify context-specific ligand-receptor interactions for cell-cell communication analysis. *Brief Bioinform* 2025;26:bbaf693. 10.1093/bib/bbaf693 PMC12741564 41451540

